# Arthroscopic Minced Cartilage Implantation for Chondral Lesion at the Glenoid in the Shoulder: Technical Note

**DOI:** 10.1016/j.eats.2024.103218

**Published:** 2024-09-10

**Authors:** Martin Bischofreiter, Christina Hraba, Franziska Lioba Breulmann, Michael Gruber, Michael Gattringer, Georg Mattiassich, Reinhold Ortmaier

**Affiliations:** a Department of Orthopedic Surgery, Ordensklinikum Linz Barmherzige Schwestern, Vinzenzgruppe Center of Orthopedic Excellence, Teaching Hospital of the Paracelsus Medical University, Salzburg, Austria; b Medical Faculty, Johannes Kepler University Linz, Linz, Austria; c Department of Orthopaedics and Traumatology, Klinik Diakonissen Schladming, Schladming, Austria; d Department of Orthopaedic Surgery, Universitätsklinik Balgrist, Zürich, Switzerland

## Abstract

Joint cartilage damage is a common condition, and various approaches exist to address these defects. Whenever conservative treatments have been exhausted or are inadequate, surgery should be taken into consideration. However, it is essential to consider the size of the damage as well as the subchondral bone involvement. As joint replacement is not an appropriate treatment for young people, a joint-preserving technique should be preferred. One option is minced cartilage implantation. This surgical procedure is appropriate for defects of around 2 cm^2^. Studies demonstrated exceptional short-term and midterm outcomes in the knee and hip. This description of technique focuses on the implementation of AutoCart augmentation on the glenoid. The purpose of this note is to gain technical evidence of an all-arthroscopic cartilage implantation technique performed within the glenoid cavity.

Articular chondral defects are common pathologies in the shoulder.^1^ The management of chondral lesions is hindered by the limited capacity to repair articular cartilage autologously.^2^ While arthroplasty is a viable treatment option in the elderly population, younger patients should be considered for preservation due to the limited life span of the prothesis.^1^^,^^3^

Most cartilage reparation methods fall into 2 main categories: those that aim to stimulate the bone marrow and cell-based techniques.^4^ Cell-based cartilage repair methods provide the highest quality of repair tissue, such as minced cartilage implantation (MCI).^4^^,^^5^ A variety of surgical techniques are used to treat cartilage damage (Fig 1).^5^ These techniques are categorized into regeneration (like autologous chondrocyte implantation), repair (such as drilling and microfracture), or replacement with allografts.^6^^,^^7^

**Fig 1:**
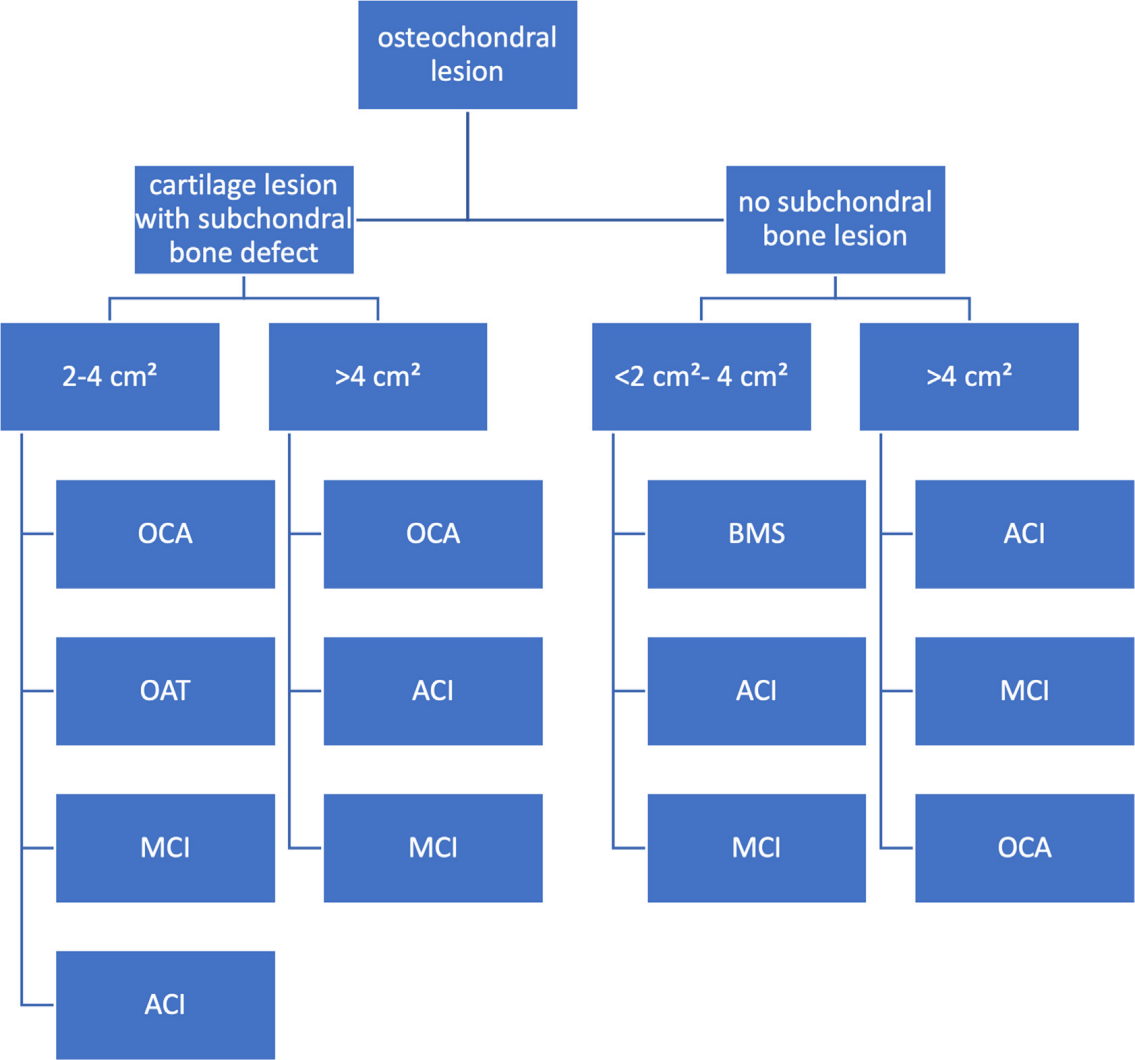
Cartilage restoration algorithm. Osteochondral lesions are divided into cartilage lesion with subchondral bone defect or no subchondral bone lesion. For lesions 2 to 4 cm^2^, osteochondral allograft (OCA), osteochondral autograft transfer (OAT), autologous chondrocyte implantation (ACI), or minced cartilage implantation (MCI) can be assessed. For lesions >4 cm^2^, OAT is more difficult and not performed. Osteochondral lesions with no subchondral bone loss can be treated with bone marrow stimulation (BMS), ACI, or MCI (only for younger patients or patients with contraindications for other procedures) if the lesion is between 2 and 4 cm^2^. If the lesion is >4 cm^2^ and without subchondral bone loss, ACI, MCI, or OCA is a possible treatment option.

MCI is a 1-stage procedure, first described in 1982, that showed early good results in studies in 2006. Unlike other procedures, it is more cost-efficient and simple and has high biological potential.^4^^,^^8^ It has already been introduced for other joints, especially for the knee, with improvement of pain and function.^9^

## Surgical Technique

MCI is typically performed arthroscopically in 1 stage,^1^ although examples of an open approach can be found.^10^ At first, arthroscopically performed minced cartilage procedures showed promising clinical outcomes.^3^ Additionally, the surgeon may choose to perform arthroscopy wet or dry. If the procedure is started wet, the arthroscopic fluid must always be removed before starting the cartilage fragment implantation.^1^ In this case, we have chosen the wet method (Video 1).

### Equipment

• Standard instrument set for arthroscopy
• 3.0-mm shaver (recommended Sabre, smooth; Arthrex)
• GraftNet (Arthrex)
• Thrombinator (Arthrex)
• Three autologous conditioned plasma (ACP) double syringes (Arthrex)
• Centrifuge (Hettich)
• Suture material, sterile swab

### Preoperative Preparation

**Fig 2:**
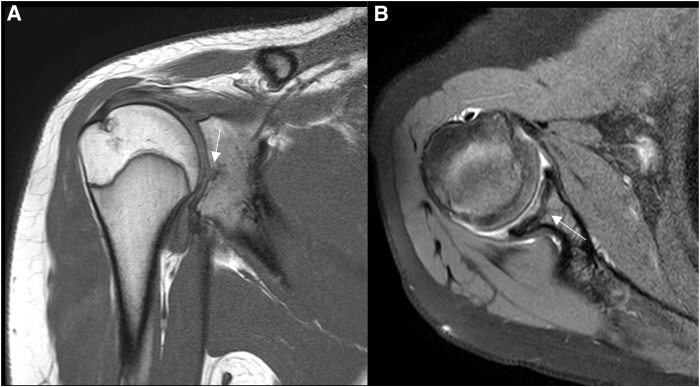
Magnetic resonance imaging of the right shoulder presurgery. An osteochondral lesion in the center of the glenoid is present. The left side shows a sagittal view in PDW-sequence, and the right side presents a transversal view in T1-sequence. The osteochondral lesion of the glenoid is demonstrated (→). It is centrally located in the glenoid.

Preoperatively, a detailed clinical assessment is conducted, including range of motion, rotator cuff strength, and impingement signs. The preoperative x-rays and magnetic resonance imaging (MRI) show the location and extent of the cartilage lesion (Fig 2).^1^

Perioperative antibiotic prophylaxis is highly recommended. To mitigate adverse effects on the platelet-rich plasma (PRP), it is advisable to obtain the blood sample under sterile conditions prior to the administration of anesthetics and antibiotics, as mentioned above.^5^^,^^11^ The required quantity of PRP is approximately 10 to 15 mL.^5^

### Intraoperative

##### Positioning and Diagnostic Arthroscopy

**Fig 3:**
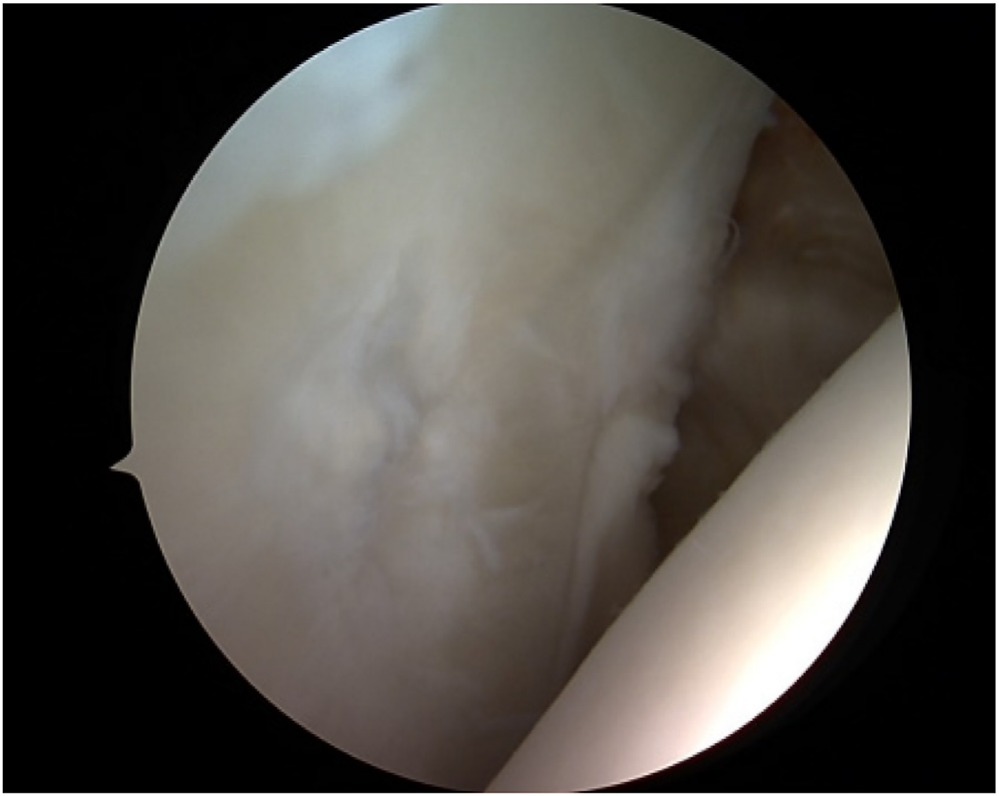
Intraoperative image demonstrating the chondral defect on the glenoid measuring 1 × 0.8 cm (→). On the right side, there is the intact cartilage of the humerus. The view is pointing from the posterior glenoid on the left side to the anterior glenoid in the middle. On the right side of the picture, the humerus is visible.

The surgical area is washed and draped under sterile conditions. The patient is positioned in a beach-chair posture with the index arm secured in an adjustable arm holder.^1^ To assess for any associated intra-articular pathology and to identify the exact location of the chondral lesion, a standardized diagnostic arthroscopy in the posterior portal is done (Fig 3).^1^^,^^5^ Before starting the AutoCart procedure (Arthrex), concomitant pathologies like rotator cuff repair should be treated.

##### Preparation of Chondral Deformation

**Fig 4:**
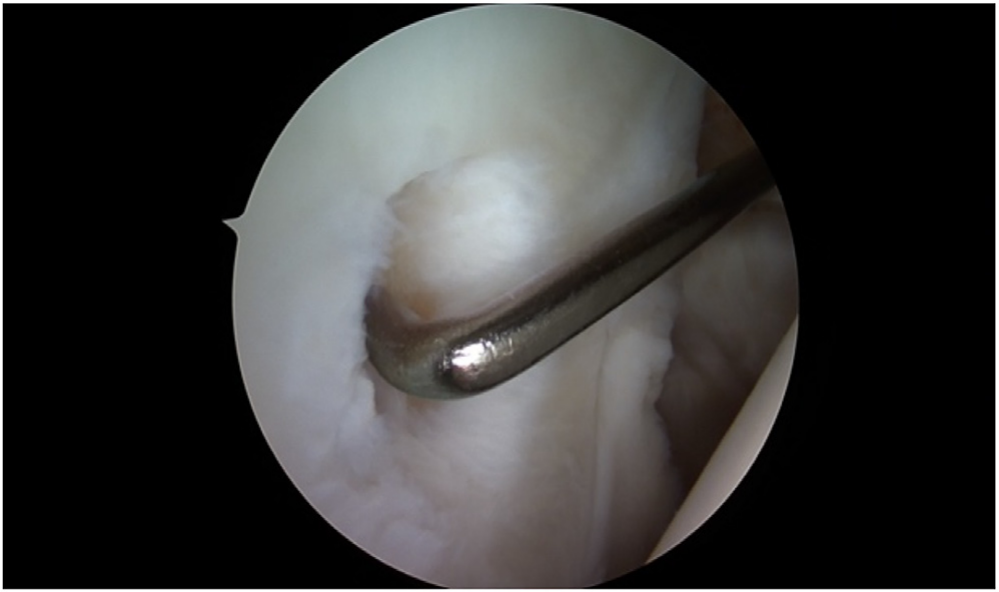
Intraoperative image of the debridement of the chondral lesion for preparation of cell implantation. Measuring of the lesion by using the hook (→). The hook is almost fully insertable into the defect. The view is posterior to anterior.

**Fig 5:**
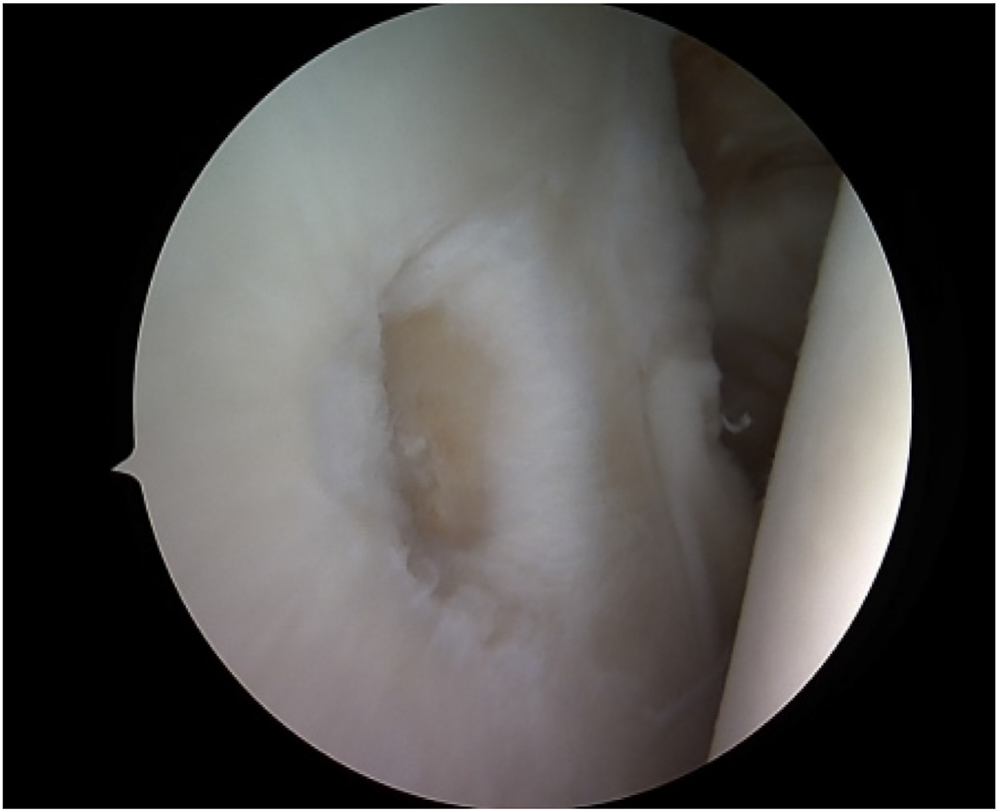
Intraoperative image demonstrating the debrided lesion after harvesting cartilage and creating vertical defect edges. The view is posterior to anterior.

Once the lesion has been localized, the site has to be prepared carefully, and suitable donor cartilage must be collected.^5^ The lesion is visualized and measured using a probing hook (Fig 3). To remove all the damaged cartilage, debridement of the chondral defect is performed.^1^^,^^11^ The cartilage can then be harvested circumferentially from the chondral walls of the defect margin (Fig 4).^1^^,^^5^ The purpose of this procedure is to preserve and harvest healthy areas of cartilage for grafting and to create vertical defect edges (Fig 5).^5^ Hence, 2 methods are accessible: first, a distinct shaving device such as the shaver Sabre (Arthrex) model equipped with 3.0-mm shaver blades can be used in conjunction with a GraftNet autologous tissue collector (Arthrex). Second, a conventional technique can be employed by using either a small-sharp-spoon or a ring curette.^1^^,^^11^ The fragments should be grasped with forceps as necessary, divided on the back table with a No. 10 or 15 blade, and finally minced with a 3.0-mm shaver blade.

##### Chondral Fragment Preparation

Cartilage fragments should always be kept moist in PRP or water during intraoperative storage and cutting. The fragment size should be 1 mm³ or less with approximately 150 chips per 1 cm^2^ of cartilage lesion (in case of a defect depth greater than 2 mm). The smaller the cartilage fragments, the greater their proliferation and the more extracellular matrix (ECM) is produced.^1^^,^^12^ The autologous PRP from the patients’ venous blood acts as a biological agent and forms a binder to secure the chips in place. A Thrombinator (Arthrex) is used to prepare the PRP. A volume of 3 mL of ACP is incorporated into the system to produce the autologous thrombin solution.^1^

To obtain the ACP, a total of 45 mL of venous whole blood is collected in 3 ACP double syringes, which are then centrifuged at 1,500 rpm for 5 minutes. The plasma supernatant is drawn off and transferred to a sterile tube. Subsequently, a volume of 3 mL of the ACP is infused into the Thrombinator, and the mixture is shaken vigorously for 5 to 10 seconds. The Thrombinator activates the coagulation cascade, causing a thrombin solution. After a 10-minute period of rest, the system is shaken to dislodge the resulting clot. A further 6 mL of ACP is now added before waiting another minute. The clot is broken up again by shaking, and the thrombin solution is removed.^13^

##### Insertion Into the Lesion

**Fig 6:**
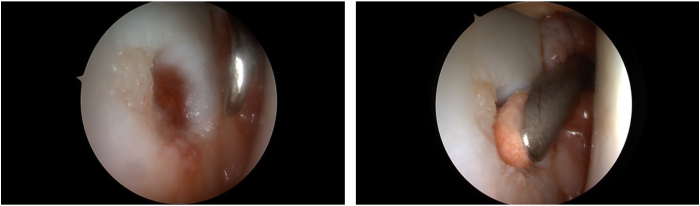
Intraoperative image presenting the lesion under dry and bloodless conditions, prepared for the chip implantation. The lesion is primary after debridement of the insufficient cartilage filled with blood. The lesion is dried with the help of a halfpipe and a swab (→). The view is posterior to anterior.

**Fig 7:**
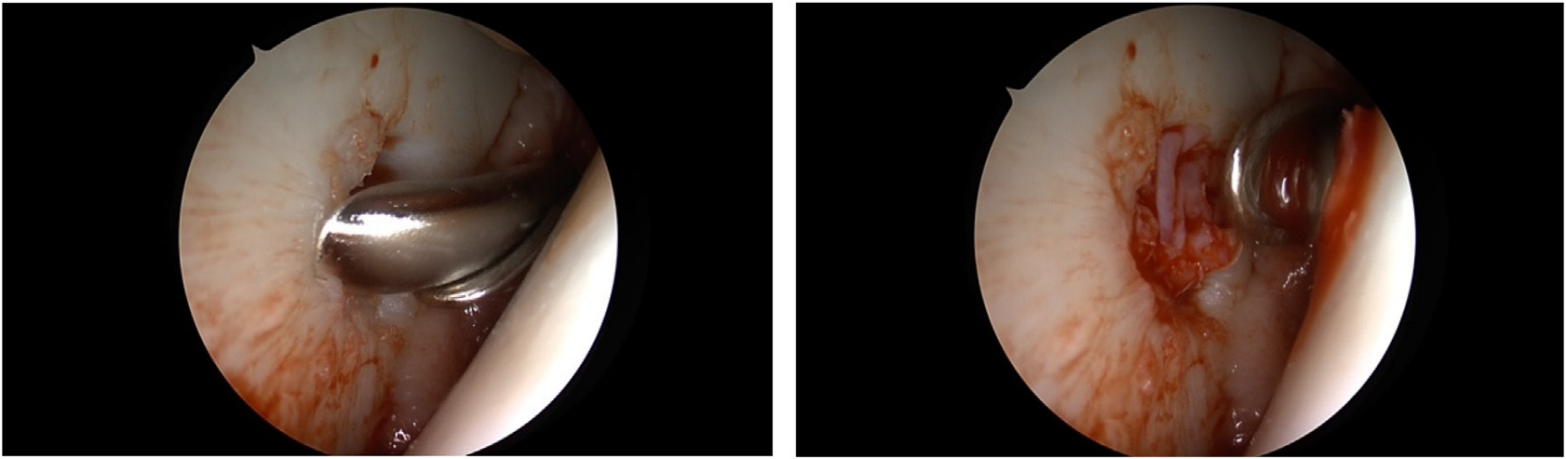
Intraoperative image displays insertion of the cell–platelet-rich plasma–mass under dry and bloodless circumstances to 80% to 90%. On the left, a cannula (→) is placed above the cartilage defect. In a second stage, on the right, the instrument is removed and the cartilage defect is visible. The view is posterior to anterior.

**Fig 8:**
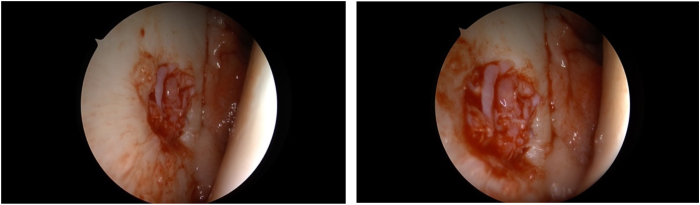
Intraoperative image shows the inserted mass into the lesion with the final layer. The cartilage defect is completely covered (→). The view is posterior to anterior.

The joint is aerated to implant the cartilage under dry and bloodless circumstances (Fig 6). The minced cartilage is combined with the PRP in a 3:1 ratio for a moldable mass. A curved application device (Tuohy curved needle; Arthrex) is filled with the cartilage/plasma mixture. The paste is gradually injected and dispersed over the lesion until 80% to 90% fill is achieved (Fig 7).^5^^,^^13^^,^^14^ The previously produced thrombin solution is applied dropwise over the cartilage paste. (Fig 8).^1^^,^^5^ Because the ACP/thrombin mixture rapidly forms a clot, this should be added quickly drop by drop to the filled defect.^1^^,^^13^ After final sealing, it is recommended to wait for 2 minutes to ensure sufficient hardening of the clot.^13^

##### Postoperative Management

After surgery, the shoulder is immobilized and placed in internal rotation in a shoulder brace for 24 hours. Wearing the brace is suggested for 4 weeks, with only passive mobilization. After that phase, a gradual increase of active movement can be started.^1^ In collaboration with the physiotherapy department, we developed an interdisciplinary aftercare program (Table 1).

**Table 1:** Postoperative Treatment and Rehabilitation Protocol

Time After Surgery	Physical Therapy Plan
1-4 weeks	• Home program for finger and elbow exercises, no active and passive movements if possible • Shoulder brace • Motor splint • Analgetic electrotherapy
5-8 weeks	• Active and passively supported movements in all directions • Muscle-stimulating electrotherapy
9-12 weeks	• Free activity • Slow increase in weightbearing in all planes • Underwater therapy
13 weeks	• Full sports load permitted

##### Possible Complications

• Completely filling or overfilling the defect can cause shear stress on the graft.^14^
• Obtaining a sufficient quantity of PRP is not possible: patients should be advised not to take any medication that may impair platelet function (e.g., acetylsalicylic acid).^13^
• If enough PRP cannot be obtained, allogenic fibrin can be used to seal the cartilage mass.^13^
• If the plasma supernatant is not stopped in time and erythrocytes are aspirating in the double-chamber syringe, the entire volume can be reinjected and the centrifugation process restarted.^13^
• A Thrombinator can be employed to prepare ACP again if the autologous thrombin solution does not adhere. Autologous fibrin glue may also be necessary.^13^
• If intra-articular hemorrhage causes impaired vision, an high-frequency applicator can be used to stop the bleeding. Adrenaline may be added to the irrigation fluid.^13^

## Discussion

MCI is a cell-based technique in which the patient’s own cartilage is harvested, fragmented, and then reimplanted.^5^ Studies on the knee and in the laboratory show promising short- and medium-term results with the use of cartilage fragments for implantation in the treatment of chondral and osteochondral defects. This may be attributed to the biological potential and activity of the chondrocyte, as well as the enhancement facilitated by PRP.^3^^,^^12^^,^^15^

Gebhardt et al.^15^ investigated the impact of the shaver tool on the biological potential of chondrocytes. The in vitro study demonstrates the possibility of using arthroscopic shavers for harvesting and grinding joint cartilage in terms of chondrocyte survival and function. It was observed that the viability of cells minced with a shaver was higher than that of cells derived from curettage. Furthermore, adding PRP did not affect cell outgrowth or cell viability in the transferred cartilage tissue. It did increase the normal proteoglycan content of the chondrocyte spheroids. This suggests that the use of PRP improves the ability of chondrocytes to produce an extracellular matrix.

Runer et al.^16^ evaluated MCI in 28 patients with chondral and subchondral lesions with a size of 3.5 ± 1.8 cm^2^ in the knee joint. The study showed good graft longevity at a 60-month follow-up, and only 1 of 28 patients needed revision surgery and no significant differences in pain or knee function at the 2-year follow-ups. There is limited evidence on the use of MCI in the treatment of cartilage defects in the shoulder. Only 1 report by Karkosch et al.^3^ from 2023 exists, which focuses on the treatment of cartilage damage in the humerus head of a 33-year-old patient and provides important short-term results. The recovery time and time to achieve full fitness were relatively short. In summary it can be stated that MCI yields positive short-term outcomes in the glenohumeral joint. However, as shown in Table 2 besides many advantages there are also some disadvantages. To obtain more relevant clinical data, further studies with larger cohorts and long-term results are required.

**Table 2:** Advantages and Disadvantages of Minced Cartilage Implantation

Advantages	Disadvantages
Completely homologous method	Outcomes partially dependent on quality of ACP
Instant access	Comorbidities need to be considered
Rapid method	Contraindications for use of ACP must be assessed
No significant tissue alteration	Potentially limited by defect size
Single-step procedure	Long-term follow-up not yet available
Low costs	Studies with larger cohorts are rare
All-arthroscopic technique	
Transplantation of chondrocytes and extracellular matrix	

ACP, autologous conditioned plasma.

## Disclosures

All authors (M.B., C.H., F.L.B., M. Gruber, M. Gattringer, G.M., R.O.) declare that they have no known competing financial interests or personal relationships that could have appeared to influence the work reported in this paper.

## Supplementary Data

Video 1 Operative video of the cartilage lesion and procedure of the minced cartilage implantation.
